# Transient Hyperglycemia and Hypoxia Induce Memory Effects in AngiomiR Expression Profiles of Feto-Placental Endothelial Cells

**DOI:** 10.3390/ijms222413378

**Published:** 2021-12-13

**Authors:** Jasmin Strutz, Kathrin Baumann, Elisa Weiss, Ursula Hiden

**Affiliations:** 1 Department of Obstetrics and Gynecology, Medical University of Graz, 8036 Graz, Austria or j.strutz@fh-kaernten.at (J.S.); kathi.baumann@chello.at (K.B.); elisa.weiss@medunigraz.at (E.W.); 2 Institute of Biomedical Science, Carinthia University of Applied Sciences, 9020 Klagenfurt, Austria; 3 Institute of Biomedical Science, Department of Health Studies, FH Joanneum University of Applied Sciences, 8020 Graz, Austria

**Keywords:** hyperglycemia, hypoxia, memory effect, feto-placental endothelial cells, angiomiR

## Abstract

Gestational diabetes (GDM) and preeclampsia (PE) are associated with fetal hyperglycemia, fetal hypoxia, or both. These adverse conditions may compromise fetal and placental endothelial cells. In fact, GDM and PE affect feto-placental endothelial function and also program endothelial function and cardiovascular disease risk of the offspring in the long-term. MicroRNAs are short, non-coding RNAs that regulate protein translation and fine tune biological processes. A group of microRNAs termed angiomiRs is particularly involved in the regulation of endothelial function. We hypothesized that transient hyperglycemia and hypoxia may alter angiomiR expression in feto-placental endothelial cells (fpEC). Thus, we isolated primary fpEC after normal, uncomplicated pregnancy, and induced hyperglycemia (25 mM) and hypoxia (6.5%) for 72 h, followed by reversal to normal conditions for another 72 h. Current vs. transient effects on angiomiR profiles were analyzed by RT-qPCR and subjected to miRNA pathway analyses using DIANA miRPath, MIENTURNET and miRPathDB. Both current and transient hypoxia affected angiomiR profile stronger than current and transient hyperglycemia. Both stimuli altered more angiomiRs transiently, i.e., followed by 72 h culture at control conditions. Pathway analysis revealed that hypoxia significantly altered the pathway ‘Proteoglycans in cancer’. Transient hypoxia specifically affected miRNAs related to ‘adherens junction’. Our data reveal that hyperglycemia and hypoxia induce memory effects on angiomiR expression in fpEC. Such memory effects may contribute to long-term adaption and maladaption to hyperglycemia and hypoxia.

## 1. Introduction

Maternal metabolic and inflammatory disorders in pregnancy such as gestational diabetes (GDM) and preeclampsia (PE) affect and shape the fetal environment. GDM causes hyperglycemia and hypoxia in the fetus [1,2,3], and also PE is associated with chronic or subchronic fetal hypoxia [4,5]. Both hyperglycemia and hypoxia are well-known disturbing factors of endothelial function: Hyperglycemia induces the production of reactive oxygen species (ROS) by the mitochondrial electron chain and generates advanced glycation end products, i.e., proteins modified by non-enzymatic glycation, which are the main underlying causes for endothelial dysfunction [6,7]. Also hypoxia and fluctuations in oxygen levels are associated with oxidative stress and increased ROS production [8], and disturb endothelial function [9].

The adverse biochemical processes induced by hyperglycemia and hypoxia are, in general, of reversible nature. However, offspring of pregnancies exposed to such conditions in utero, i.e., after pregnancies complicated by maternal GDM or PE, possess an increased risk to develop endothelial dysfunction and cardiovascular diseases later in life [10,11]. This fetal programming is also associated with epigenetic changes. For instance, altered DNA methylation has been observed in feto-placental endothelial cells after GDM pregnancy [12] and in neonatal endothelial progenitor cells after PE pregnancies [13]. Underlying causes are multifaceted, but long-lasting effects of hyperglycemia and hypoxia play a role.

In fact, it has been well established that the negative effect of hyperglycemia on endothelial function continues even after the establishment of normoglycemia [14,15]. This phenomenon of transient exposure to hyperglycemia has been termed ‘glycemic memory’ or ‘hyperglycemic memory’ and may be explained in part by persistent epigenetic changes caused by hyperglycemia. Hyperglycemic memory can also be observed in vitro after exposure of endothelial cells to hyperglycemia, and includes epigenetic mechanisms, such as DNA methylation changes [16,17] as well as altered histone methylation [18,19].

Hyperglycemia is not the only condition inducing long-term effects after a transient exposure. A hypoxic memory has been identified in kidney disease, induced by acute kidney injury, which also involves DNA methylation and histone modifications [20]. In endothelium, a hypoxia-induced memory effect has not yet been established, however, the fact that HIF-1 stability and activity as well as ERK signalling differ after acute vs. transient hypoxia [21,22] highlights the distinct effects of them. Moreover, the recently discovered finding that hypoxia alters histone demethylases in endothelial cells [23] potentially enables long-term changes in gene expression and suggests a hypoxic memory in endothelial cells as well.

MicroRNAs are short, non-coding RNA molecules that bind complementary to target messenger RNAs (mRNAs), and thus, interfere with translation. So far, about 2600 miRNAs have been described in humans, with in total more than 380,000 calculated interactions with target mRNAs (miRTarBase database [24]). Like mRNAs, miRNA expression is regulated at different levels, i.e., by transcription factors, DNA methylation, and histone modification. Whilst many miRNAs possess their own regulatory elements, other miRNAs are located and co-expressed with the host gene, and thus, regulated by the host gene promoter [25]. This means that miRNA expression can be regulated in the short and long-term, and also through programming mechanisms. MiRNAs act as critical fine tuner in virtually all biological processes. For instance, a group of about 20 miRNAs is particularly involved in the regulation of endothelial function and angiogenesis, and these miRNAs have been termed ‘angiomiRs’ [26,27]. miRNAs are also implicated in memory effects. miRNAs-mediated modulation of gene expression was identified to participate in immune memory [28], and altered miRNA expression occurs during glycemic memory of the heart [29] and in endothelial cells [30].

This raises the questions whether both periods of transient hyperglycemia as well as transient hypoxia may alter angiomiR expression in the endothelium of the fetal vasculature and whether the effects of transient stimuli differ from effects of current stimuli, i.e., effects during the treatment.

To this end, we isolated primary feto-placental endothelial cells (fpEC) from placental arteries. Placental vessels are part of the fetal vasculature and exposed to the fetal blood stream. Thus, fpECs experience the same environmental stimuli as fetal endothelial cells and serve as a model for fetal endothelium. FpEC were isolated after normal pregnancy, i.e., after a pregnancy without pathological hyperglycemic/hypoxic periods. The cultured cells were under hyperglycemic or hypoxic conditions for 3 days, followed by reversal to normoglycemic/normoxic conditions for another 3 days. AngiomiR profiling was performed immediately after the exposure to hyperglycemia and hypoxia to determine the direct effect of the stimuli. Moreover, analysis was performed 72 h after return to normal conditions to investigate the transient effects.

## 2. Results

**Figure 1:**
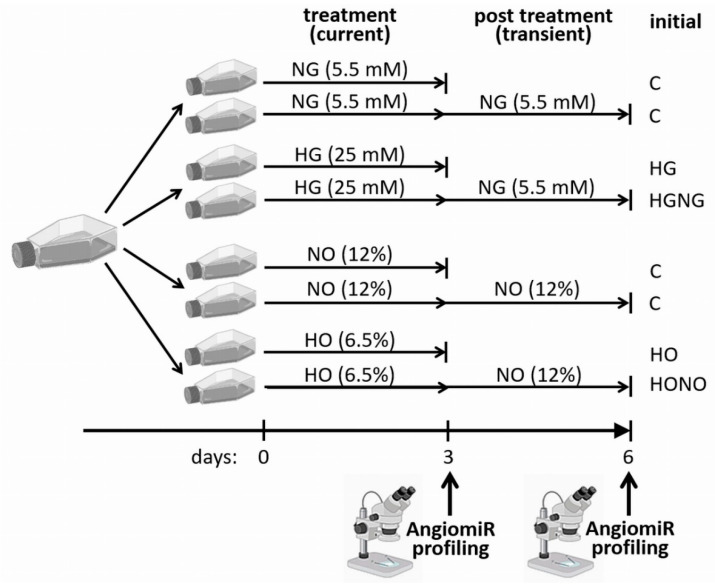
Experimental setup. Hyperglycemic treatment was performed with n = 10 individual fpEC isolations; hypoxic treatment was performed with n = 9 individual fpEC isolations. C: control condition; NG: normoglycemia (5.5 mM glucose); HG: hyperglycemia (25 mM glucose); NO: normoxia (12% O_2_); HO: hypoxia (6.5% O_2_); HGNG: transient hyperglycemia with reversal to normoglycemia; HONO: transient hypoxia with reversal to normoxia.

The experimental setup to analyse and distinguish current vs. transient effects of hyperglycemia and hypoxia on angiomiR expression in fpEC is illustrated in Figure 1.

### 2.1. Hyperglycemia and Hypoxia Did Not Alter Microscopical Growing Pattern of fpEC

**Figure 2:**
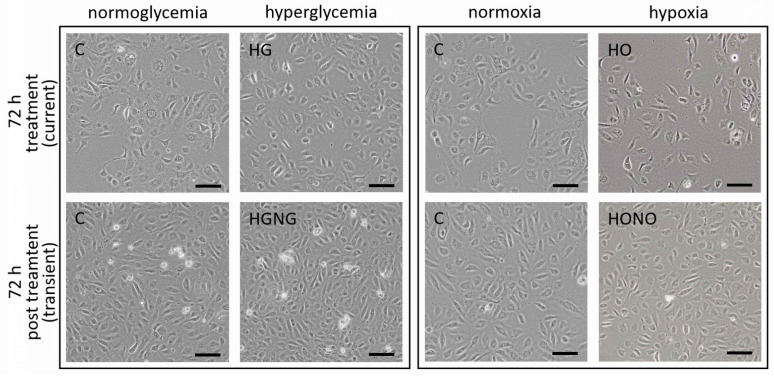
Microscopic morphology and growing pattern of fpEC. Representative pictures of cells cultured at current and transient hyperglycemia (HG; HGNG, **left** panel) and hypoxia (HO; HONO, **right** panel) and at control conditions. Scale bar: 100 µm.

Exposure of fpEC to current hyperglycemia (HG) and hypoxia (HO) for 72 h did not alter the cell shape or growing pattern of fpEC. Also after reversal to control conditions (HGNG and HONO, respectively), the growing pattern was similar to the cells grown under control conditions continuously (Figure 2).

### 2.2. Current and Transient Effects of Hyperglycemia and Hypoxia on AngiomiRs

For expression profiling of angiomiRs, two distinct normalization strategies were followed in parallel: First, miRNAs were normalized to the geometric mean of five small non-coding housekeeping RNAs, i.e., RNU6-2; miR-28-3p; miR-30b-5p; miR-191-5p; and miR-423-3p. Second, in order to identify angiomiRs that stand out in their regulation from the collective of the 19 angiomiRs, each miRNA was normalized to the geometric mean of the total of angiomiRs. Both strategies revealed very similar results and overall expression pattern, with only minor differences in significance levels.

**Figure 3:**
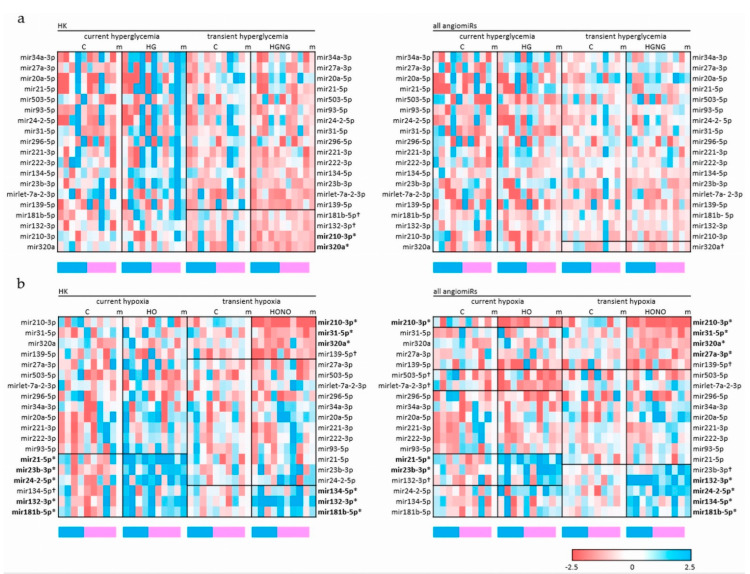
Heatmap illustrating the effect of current and transient treatments on angiomiR expression. The changes in angiomiR expression under current and transient hyperglycemia (HG; HGNG) vs. control conditions (**a**) and changes in angiomiR expression under current and transient hypoxia (HO; HONO) vs. control conditions (**b**). ‘HK‘ indicates that miRNAs were normalized to the geometric mean of five housekeepers; ‘all angiomiRs‘ indicates that miRNAs were normalized to the geometric mean of all angiomiRs. Downregulation vs. respective control condition is highlighted by red color, upregulation by blue color, and unchanged expression by white color. The columns entitled with ‘m’ represent the mean value of the condition. * indicates that the angiomiR is significantly different from control condition (*p* < 0.05), ^†^ indicates that angiomiR differs from control conditions by trend (*p* < 0.1). Groups of angiomiRs that are changed significantly or per trend are framed with a line. miRNAs are printed in bold if they are significantly changed by any condition (current or transient) in the respective heatmap. The blue (male) and pink (female) bars underneath the heatmaps indicate the sex of the fpEC donor. The respective color codes for up- and downregulation are given in the lower right corner. Heatmaps were generated using MS Excel.

Expression analysis revealed that current hyperglycemia (HG) for 72 h had no effect on angiomiRs, regardless of whether angiomiRs were normalized to housekeepers (HK) or to total angiomiRs. Also, the reversion of cells to normoglycemia (HGNG) affected the expression of angiomiRs only slightly: After normalization to HK, two angiomiRs were significantly altered whilst normalisation to total angiomiRs revealed no significant changes in angiomiR profile (Figure 3a, Table 1).

In contrast to current hyperglycemia, current hypoxia (HO) had a more pronounced effect on angiomiR expression: HO significantly altered the expression of miR-132-3p; miR-181b-5p; miR-21-5p; miR-23b-3p; and miR-24-2-5p when normalized to housekeeping genes (HK) and of miR-21-5p, miR-210-3p, and miR-23b-3p when normalized to total angiomiRs (Figure 3b, Table 1).

Reversal of cells to normoxic conditions revealed that transient hypoxia (HONO) changed the expression of more angiomiRs than current HO did: normalisation to HK revealed six significantly altered angiomiRs (miR-132-3p, miR-134-5p, miR-181b-5p, miR-210-3p, miR-31-5p, and miR-320a), and normalisation to total angiomiRs identified the same set of angiomiRs with two additionally altered miRNAs, i.e., miR-27a-3p and miR-24-2-5p (Figure 3b, Table 1).

In previous studies, we observed sex differences in miRNA expression of fpEC, and thus, all analyses were also performed in consideration of fetal sex (not shown). In our small cohort with 5/5 and 5/4 fpEC of male vs. female progeny, we did not detect any sex-specific difference in angiomiR regulation. Fetal sex is indicated below the heatmaps in Figure 3 as a blue (male) or pink (female) box.

### 2.3. Current and Transient Effects of Hypoxia on AngiomiR-Regulated Functional Pathways

Because of the absent and little effect of current and transient hyperglycemia on angiomiR expression, no pathway analysis was performed. For pathway analysis of current and transient hypoxia effects, in addition to significantly altered miRNA, we also included miRNAs that were altered by trend (*p* < 0.1). There are several software tools for miRNA pathway analysis that are based on distinct algorhythms and databases. We employed three different software tools, i.e., DIANA miRPath v.3 [31], MIENTURNET [32], and MiRPathDB 2.0 [33], and interlinked the results by selecting only pathways that were identified by at least two of the applied softwares (Table 2).

**Figure 4:**
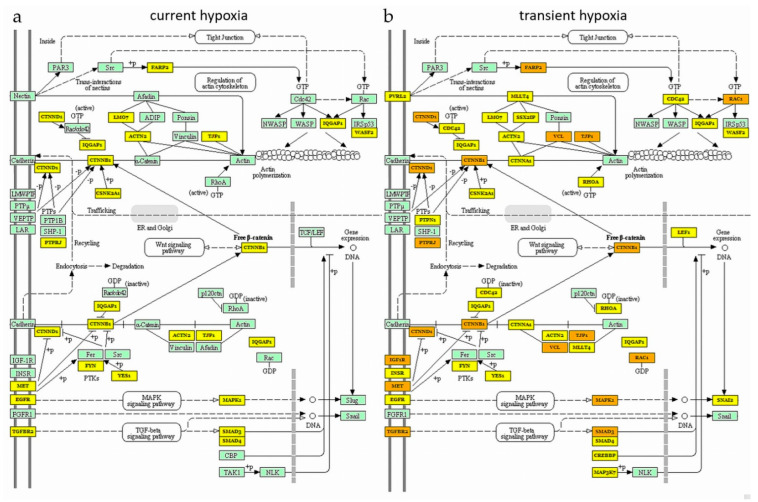
AngiomiR target mRNAs within the KEGG pathway ‘adherens junction’. This pathway was identified to be altered after transient hypoxia (HONO), but not by current hypoxia (HO). (**a**) indicates the target genes of angiomiRs altered (significantly or by trend) under current hypoxia (HO). (**b**) indicates the target genes of angiomiRs altered (significantly or by trend) under transient hypoxia (HONO). Yellow coloring of genes indicates that mRNAs are targeted by one miRNA, orange coloring of genes indicates mRNAs targeted by several miRNAs. KEGG pathways were generated using DIANA mirPath software.

Independently of the normalization strategy used for angiomiR profiling, current hypoxia altered miRNAs that could be mainly assigned to the functional pathways ‘Proteoglycans in cancer’ and ‘FOXO signaling pathway’. Transient hypoxia altered the pathways ‘Proteoglycans in cancer’, ‘HIF-1 signaling pathway’ and ‘adherens junction’. The pathway ‘adherens junctions’ was regulated by transient hypoxia only. In order to visualize this difference, we employed DIANA miRPath software to generate KEGG pathways based on angiomiRs altered by current hypoxia (HO) vs. transient hypoxia (HONO). Indeed, more target mRNAs were present, i.e., highlighted by yellow or orange, within the pathway generated, with the list of altered angiomiRs under transient hypoxia (HONO) when compared to the pathway generated with the list of altered angiomiRs under current hypoxia (HO) (Figure 4).

**Figure 5:**
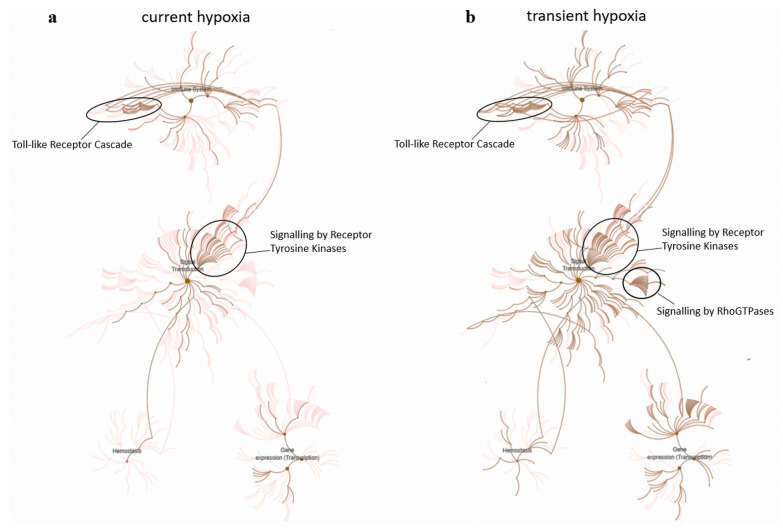
Pathway analysis of angiomiR target genes using Reactome analysis. (**a**) AngiomiRs regulated under current hypoxia either significantly or by trend were used for miRNA-target enrichment using MIENTURNET software followed by Reactome pathway analysis of biomolecular pathways. Pathways in pink are moderately overrepresented, whilst pathways in brown are highly overrepresented. Particularly ‘signalling by receptor tyrosine kinases’ (signal transduction) and ‘toll-like receptor cascade’ (immune system) were altered. (**b**) AngiomiRs regulated under transient hypoxia either significantly or by trend included similar pathways, with additional overrepresented regulation of ‘signalling by RhoGTPases’.

To generate an overview on biomolecular pathways affected by angiomiRs altered by current vs. transient hypoxia, we used an analysis tool developed for pathway analysis of mRNA lists, i.e., Reactome pathway analysis [34]. For this purpose, we generated a list of angiomiR target mRNAs for each condition using miRNA-target enrichment analysis of MIENTURNET software to create Reactome pathways. Reactome pathway diagrams display those pathways that are overrepresentatively regulated. In both conditions, i.e., current and transient hypoxia, particularly ‘signalling by receptor tyrosine kinases’ (cluster ‘signal transduction’) and ‘toll-like receptor cascade’ (cluster ‘immune system’) were overrepresented. In concordance with the previous miRNA pathway analysis, there was a stronger target mRNA overrepresentation in ‘RhoGTPas signalling’ after transient hypoxia (HONO) as when compared to current hypoxia (HO). This biomolecular pathway is closely related to the KEGG pathway ‘adherens junction’ with various overlapping molecules (Figure 5).

## 3. Discussion

In this study, we investigated the effect of current vs. transient hyperglycemia and hypoxia on angiomiR expression in fpEC. AngiomiR profiling revealed that both transient hyperglycemia and transient hypoxia altered angiomiRs, thus inducing a memory effect in angiomiR expression under both conditions. Moreover, the effect of hypoxia (current and transient) was stronger than the effect of hyperglycemia. Interestingly, some miRNAs, i.e., miR-210-3p and miR-320a, were regulated under both conditions, i.e., by transient hypoxia and transient hyperglycemia.

Transient and hence, memory effects of both hyperglycemia and hypoxia, were more pronounced than acute effects of current treatment. For instance, despite the well-known adverse effects of high glucose on endothelial function in vivo and in vitro [35,36], angiomiR pattern was unaffected by current hyperglycemia, with no change in expression profile. Several scenarios are possible that could explain the non-existent effect of high glucose on angiomiRs. First, the immediate response of angiomiRs to hyperglycemia may happen more rapidly than monitored here, already flattening after 72 h exposure. For instance, upregulation of miR-21 by hyperglycemia in human glomerular endothelial cells peaks after 48 h and declines thereafter [37]. However, the fact that hyperglycemia induced endothelial dysfunction is a long-term complication of diabetes and clinically develops over time [14,15,38], and transient intermittent hyperglycemia in diabetic patients is an independent risk factor for cardiovascular diseases [39]. This is paralleled by the in vitro finding that the effect of transient hyperglycemia on global miRNA expression of human aortic endothelial cells was greater than the effect of current hyperglycemia [30]. Absent current effect after 72 h of hyperglycemia may thus reflect the slow and long-term development of endothelial dysfunction in diabetes.

In contrast to the absent effect of current hyperglycemia on angiomiRs, current hypoxia altered six angiomiRs significantly. As every cell in the body requires oxygen for functioning, decreased oxygen immediately activates a signalling cascade that triggers a variety of biological processes, and miRNAs are involved in this cascade [40]. MiRNAs induced by hypoxia have been termed hypoxamiRs [41], and these molecules fine tune hypoxia-induced cellular adaption including apoptosis, survival, proliferation, inflammation, metabolism, and angiogenesis [41,42]. Indeed, all angiomiRs that were found significantly regulated by moderate current hypoxia have been identified as angiomiRs previously, i.e., miR-21, miR-23b, miR-24, miR-132, miR-181b, and miR-210 [42,43]. In line with this, except for miR-210-3p that was downregulated, all of them were upregulated by current hypoxia in fpEC. The fact that miR-210-3p, as a classical hypoxamiR, was reduced after 72 h of hypoxia is surprising, and we can only speculate that the induction of miR-210-3p by hypoxia has—as well as the cellular adaption to hypoxia in general [44]—a shorter dynamic than the time interval investigated here. We aimed to mimic chronic moderate hypoxic episodes, and thus, upregulation of miR-210-3p may have occurred before angiomiR analysis at 72 h. Interestingly, in rats, a downregulation of miR-210-3p was shown to enhance the expression of DNA repair molecules [45]. Reduction of miR-210-3p after hypoxia, i.e., in a situation of oxidative stress, may thus help counteract DNA damage.

Also transient hypoxia altered more angiomiRs (8) than current hypoxia (6), with an overlap of four commonly regulated angiomiRs. The transient effect of hypoxia in vitro, i.e., a hypoxic memory, is less investigated than hyperglycemic memory, however, in vitro data show that HIF-1 (hypoxia inducible factor 1) stability and activity differ, depending on whether cells are exposed to acute, chronic, or intermittent hypoxia (reviewed by [22]). Our data add to these findings and suggest that the difference in HIF-1 signal transduction of acute vs. transient hypoxia is paralleled by altered angiomiR profiles.

Both angiomiRs that were downregulated by transient hyperglycemia, i.e., miR-210-3p and miR-320a, were also downregulated under transient hypoxia. This overlap points to a certain, common feature between hyperglycemia and hypoxia to underlie this expression change. Indeed, both conditions lead to ROS production, and consequently, to oxidative stress [6,7,8], which could, as a hypothesis, be a common regulator of long-term reduction of miR-210-3p and miR-320a.

Also miR-320a was downregulated by both transient hyperglycemia and transient hypoxia. MiR-320a is reduced during pathogenesis of diabetic retinopathy [46], and high glucose induces a downregulation of miR-320a in human islets [47], highlighting the susceptibility of miR-320 to the diabetic environment and the role in diabetes-associated endothelial dysfunction.

Memory effects refer to changes that persist after a transient exposure to a stimulus. In in-vitro experiments, cells are usually exposed to the condition once, and the induced memory effects are observed for a relatively short period of time, i.e., several days [16,17,18,19]. Also the transient effects on miRNA expression observed in our study do represent memory effects. However, whether the changes remain long-term enough to contribute to a programming effect in vivo is unknown. Noteworthy, in a recent study we investigated the effect of GDM on global miRNA expression of fpEC [48]. Although the long-term effects of GDM on endothelial cells are composed of a buzz of many individual factors, and hyperglycemia and hypoxia are only parts of the diabetic milieu, we found two angiomiRs that were regulated (significantly and by trend) by GDM exposure and by exposure to transient hypoxia, i.e., miR-134-5p and miR-139-5p. Additionally, a novel study of He et al. used our global miRNA [48] and mRNA analyses [12] of fpEC exposed to GDM to generate miRNA-mRNA regulatory networks, and identified a network around miR-139-5p to be altered by GDM [49]. Interestingly enough, this network was also altered in the fetal heart of a streptozotocin-induced pregestational diabetes mouse model, which was also characterized by an increase in cardiac wall thickness [49]. This overlap speaks for a link between in vitro memory effects of hyperglycemia and in vivo programming by diabetes.

Pathway analysis of current vs. transient effects of hypoxia showed that, in line with an overlap of regulated angiomiRs, the pathway ‘Proteoglycans in cancer’ was significantly regulated under both conditions, i.e., current and transient hypoxia. In fact, it has been well established that the pool of proteoglycans secreted by endothelial cells is sensitive to oxygen tension [50]. The pathway ‘adherens junctions’ was overrepresented only under transient hypoxia. In line with this finding, Aslam et al. reported failure of the endothelial barrier after transient hypoxia [51], which involves adherens junction assembly. Moreover, Rho GTPases are key regulators of the endothelial barrier [52], and Rho GTPase signalling was overrepresented in reactome pathway analysis.

We see it as strength of our study that we employed two distinct normalisation methods for angiomiR profiling. MiRNA normalisation is challenging as no general housekeeping miRNAs exist. Thus, the combination of several normalizers is most appropriate when using reference genes [53]. Besides that, a global normalisation, i.e., normalisation to the mean expression level of all transcripts, was postulated as the most reliable method [53]. Our method of normalizing expression data to the mean of all angiomiRs investigated is not a global normalization, but it enables expression analysis without the use of reference genes and illustrates how individual angiomiRs behave with respect to total angiomiRs. In fact, both normalization strategies have revealed very similar results, thus confirming the expression data, and show furthermore, that hypoxia, one of the strongest mediators of endothelial function and angiogenesis, targets only certain angiomiRs.

A further strength is the employment of three different pathway analysis tools and the interlinking of their results. In contrast to mRNA pathway analysis, which integrates mRNA lists to pathways from Gene Ontology and KEGG database, miRNA pathway analysis comprises a second analysis step, i.e., the determination of target genes that use one of the several available miRNA target prediction databases, such as TargetScan or miRTarBase. As a consequence, the set of significantly altered pathways identified by the software differs. We believe that the selection of only pathways that were identified by at least two of the software tools is a method to obtain as reliable results as possible.

We are aware that our data comprise information of a small subset of miRNAs only, and prediction on long-term effects on endothelial function is difficult. Thus, the translation of the results to the overall effect of the treatments as well as to the overall behaviour of the cells should be viewed with caution. However, although angiomiRs are a small group of miRNAs, they are significantly responsible for endothelial cell function, and in this sense, they are a representative parameter of endothelial cell function and dysfunction.

Moreover, although fpEC are cells of the fetal vasculature, we acknowledge the fact that they do not originate from the actual body of the fetus. However, as we do not have access to human fetal endothelial cells, fpEC represent one of the closest models possible.

Our study revealed that angiomiRs may play a role in modulation of fetal and placental endothelial function by hyperglycemia and hypoxia. In addition to the current effect of these stimuli, our data highlight that the effects of transient exposure to high glucose or hypoxia, particularly if recurrent, may have strong implications on the long-term adaption of the cells to environmental conditions. The strong response to transient stimuli indicates a large plasticity and adaption of these endothelial cells to metabolic and inflammatory factors, and memory effects may reflect a first step towards programming of endothelial function.

## 4. Materials and Methods

### 4.1. Sample Collection

Ethical approval was obtained from the Medical University of Graz (approval reference number 29-319 ex 16/17). Placentas were collected after healthy full term pregnancies, and all women provided written informed consent.

### 4.2. Isolation of Human Feto-Placental Endothelial Cells (fpEC)

Arterial fpEC were isolated from the chorionic plate as described previously [54,55]. Endothelial cell growth medium supplemented with the MV Kit was used for cell cultivation (PromoCell, Heidelberg, Germany) at 37 °C, 12% O_2_, and 5% CO_2_ in a 95% humidified incubator. All culture dishes were pre-coated with 1% porcine skin gelatin (Merck, Darmstadt, Germany).

### 4.3. Treatments

Experimental setup is illustrated in Figure 1. For hyperglycemic treatment, fpEC (n = 10 individual cell isolations) were seeded in four 75 cm^2^ cell culture flasks (3 × 10^5^ cells per flask), two in normoglycemic (5.5 mM D-glucose) and two in hyperglycemic (20 mM D-glucose) medium. The unmodified PromoCell medium served as control condition, whereas 14.5 mM D-Glucose (Merck) was added to the hyperglycemic medium. After 72 h, fpEC from one flask of the normoglycemic treatment (C) and fpEC from one flask of the hyperglycemic treatment (HG) were washed with 10 mL cold PBS (Medicago AB, Uppsala, Sweden), harvested in 700 µL of Qiazol^®^ (Qiagen, Hilden, Germany) using a cell scraper, vortexed, and stored at −80 °C for later miRNA isolation. The second flask of cells exposed to hyperglycemia was returned to normoglycemia (HGNG), and the cells of the second flask grown under normoglycemia remained at normoglycemic conditions (C). Cells were cultivated for another 72 h and harvested as described above.

For the hypoxic treatment, fpEC (n = 9 individual cell isolations) were seeded in four 75 cm^2^ cell culture flasks (4 × 10^5^ cells per flask). Two flasks were cultivated at 12% O_2,_ which reflects the umbilical artery oxygen concentration at term of gestation [56]. GDM and PE are associated with a reduction of fetal oxygen levels by 15% [2] and 32% [4], respectively. Thus, we used the oxygen concentration of 6.5% O_2_ for the cultivation of the other two flasks, which represents a reduction by 45% to mimic the moderate hypoxia of GDM and PE in vitro. Cell culture at defined oxygen concentration was performed in a customized Xvivo X3 hypoxic workstation (BioSpherix, Parish, NY, USA) at 37 °C, 5% CO_2,_ and 95% humidity. After 72 h, one flask of the normoxic condition (C) and one flask of the hypoxic condition (HO) were harvested as described above. The second flask of the hypoxic condition was switched back to normoxia (HONO) for another 72 h. The second flask of the control condition remained at normoxia (C) for another 72 h. Then, cells were harvested.

### 4.4. Cell Imaging

Cell morphology and density were observed at day 3 and day 6 for both experiments. All flasks were imaged prior to harvesting using the EVOS XL Core Cell Imaging System (Thermo Fisher Scientific, Bothel, WA, USA). For hypoxia experiments, the images were taken in the customized Xvivo X3 hypoxic workstation, equipped with the EVOS XL Core Cell Imaging System, in order to avoid a change of oxygen concentrations.

### 4.5. MiRNA Isolation and cDNA Synthesis

Total RNA enriched with miRNAs was isolated using miRNeasy Mini kit (Qiagen) according to the manufacturers’ instructions. RNA was eluted in 30 μL RNase free water, and total RNA concentration was determined using QIAxpert UV-VIS spectrophotometer (Qiagen). The content profiling mode of the QIAxpert revealed high RNA purity for all extracted samples.

For cDNA synthesis, 1 μg of total RNA was transcribed using the miScript II RT Kit (Qiagen) according to the manufacturers’ protocol. Each reaction contained 5 μL of RNA (200 ng/µL), and reverse transcription was performed for 60 min at 37 °C followed by inactivation of miScript reverse transcriptase for 5 min at 95 °C in a Mastercycler pro^®^ (Eppendorf, Hamburg, Germany). Prior to RT-qPCR, cDNA was stored at −20 °C.

### 4.6. RT-qPCR for AngiomiR Expression

Twenty angiomiRs, as obtained from the literature (Table 3), were quantified using RT-qPCR with specific miScript Primer-Assays and miScript SYBR^®^ Green PCR Kit (Qiagen). cDNA was diluted to a concentration of 0.5 ng/µL, and the PCR reactions were set up automatically in 384-well plates by using the Hamilton ID STARlet pipetting robot (Hamilton Robotics, Reno, NV, USA) with a final cDNA concentration of 2 ng in a total reaction volume of 10 µL. All reactions were performed in triplicates. RT-qPCR was carried out in the CFX384 Real-Time PCR Cycler (Bio-Rad Laboratories, Hercules, CA, USA) according to the manufacturers‘ recommendations. PCR efficiency of all primer assays was tested using a five-point standard curve with a range from 25 to 0.04 ng/reaction of pooled cDNA in triplicates. Expression of angiomiRs was relatively quantified (2^−^^ΔΔ^^C^^t^ method) [57] using two distinct strategies. The expression of miR-199a-3p was very low and below the detection limit in 14% of samples, and thus, was excluded from the analysis. First, relative miRNA expression was normalized to the geometric mean of the Ct values of five small non-coding RNAs and miRNAs, respectively, that were selected as housekeeping RNAs (RNU6; miR-28-3p; miR-30b-5p; miR-191-5p; miR-423-3p) based on the literature [58,59,60,61] and based on their stable Ct values in the experiment.

In the second strategy, we normalized the expression of each angiomiR against the geometric mean of all analyzed angiomiRs. The fold change (FC) was determined by calculating the 2^−ΔΔCt^ value of each control condition relative to the treatments and used for the generation of heatmaps in MS Excel.

### 4.7. Pathway Analysis

Functional pathways targeted by the altered miRNAs were analysed using three different tools: DIANA miRPath v.3 [72], MIENTURNET [73], and MiRPathDB 2.0 [74]. Pathways that were identified to be significantly regulated by at least two software tools were selected.

In order to display biomolecular processes with overrepresented target genes, we employed Reactome pathway database. The list of genes targeted by the altered miRNAs was obtained from MIENTURET using miRNA-target enrichment analysis based on miRTarBase database. KEGG pathways targeted by the set of miRNAs were generated using DIANA miRPath v.3.

### 4.8. Statistical Analysis

Data analysis was performed using GraphPad Prism Software Version 9.1.0 and IBM SPSS Statistics 26. For statistical analysis, ΔCt values were used for paired *t*-test (based on individual cell isolations). For generation of heatmaps and comparison of expression levels, 2^−ΔΔCt^ values (mean ± SD) were used, either based on the geometric Ct mean of the housekeeping miRNAs, or based on the geometric Ct mean of all angiomiRs analyzed. A *p*-value < 0.05 was considered as statistically significant; a *p*-value < 0.1 was regarded as a trend.

## Figures and Tables

**Table 1:** AngiomiR expression changes after current exposure to hyperglycemia (HG) or hypoxia (HO) and after reversal to control conditions (HGNG and HONO, respectively).

		Normalized to HK		Normalized to all angiomiRs	
		HG mean ± SD	HGNG mean ± SD	HG Mean ± SD	HGNG mean ± SD
**Exposure to current and transient hyperglycemia**	let-7a-2-3p	1.09 ± 0.50	0.68 ± 0.23	0.83 ± 0.55	0.89 ± 0.41
	**mir132-3p**	1.02 ± 0.34	**0.74 ± 0.15 ** ^†^	0.94 ± 0.31	0.89 ± 0.16
	mir134-5p	1.01 ± 0.46	0.85 ± 0.24	0.92 ± 0.23	0.88 ± 0.11
	mir139-5p	1.49 ± 1.43	0.68 ± 0.19	0.84 ± 0.67	0.78 ± 0.30
	**mir181b-5p**	1.45 ± 1.05	**0.82 ± 0.17 ** ^†^	0.89 ± 0.24	0.93 ± 0.16
	mir20a-5p	1.41 ± 1.54	1.16 ± 0.59	1.11 ± 1.04	1.33 ± 0.49
	**mir210-3p**	1.21 ± 1.34	0.68 ± 0.14 **	0.77 ± 0.46	0.84 ± 0.25
	mir21-5p	1.40 ± 1.04	0.81 ± 0.37	1.04 ± 0.67	0.91 ± 0.33
	mir221-3p	1.19 ± 0.80	0.79 ± 0.34	1.14 ± 0.44	0.96 ± 0.36
	mir222-3p	1.13 ± 0.67	0.79 ± 0.20	1.01 ± 0.37	1.09 ± 0.30
	mir23b-3p	1.40 ± 0.71	0.77 ± 0.23	1.09 ± 0.52	0.77 ± 0.23
	mir24-2-5p	1.17 ± 0.96	0.88 ± 0.48	0.80 ± 0.57	1.01 ± 0.42
	mir27a-3p	1.54 ± 0.87	0.90 ± 0.33	1.02 ± 0.44	1.05 ± 0.26
	mir296-5p	1.02 ± 0.89	0.91 ± 0.45	1.07 ± 0.87	0.91 ± 0.45
	mir31-5p	1.16 ± 0.86	0.94 ± 0.45	0.77 ± 0.48	1.07 ± 0.34
	**mir320a**	1.01 ± 0.39	0.65 ± 0.12 *	0.85 ± 0.41	**0.83 ± 0.21 ** ^**†**^
	mir34a-3p	1.65 ± 1.10	0.87 ± 0.41	1.27 ± 0.67	1.01 ± 0.35
	mir503-5p	1.38 ± 1.05	0.88 ± 0.49	0.75 ± 0.46	1.10 ± 0.68
	mir93-5p	1.29 ± 0.92	0.82 ± 0.26	0.90 ± 0.31	0.99 ± 0.18
		**Normalized to HK**		**Normalized to all angiomiRs**	
		**HO mean ± SD**	**HONO mean ± SD**	**HO mean ± SD**	**HONO mean ± SD**
**Exposure to current and transient hypoxia**	**let-7a-2-3p**	0.85 ± 0.34	1.19 ± 0.54	**0.61 ± 0.27 ** ^**†**^	1.15 ± 0.39
	**mir132-3p**	1.95 ± 1.45 *	1.83 ± 0.55 **	**1.47 ± 0.73 ** ^**†**^	1.83 ± 0.65 **
	**mir134-5p**	**1.60 ± 1.42 ** ^**†**^	1.34 ± 0.61 *	1.09 ± 0.69	1.41 ± 0.66 *
	**mir139-5p**	1.15 ± 1.03	**0.66 ± 0.35 ** ^**†**^	0.85 ± 0.83	**0.70 ± 0.35 ** ^**†**^
	**mir181b-5p**	1.71 ± 1.06 *	1.34 ± 0.44 *	1.21 ± 0.53	1.36 ± 0.38 *
	mir20a-5p	1.33 ± 0.60	1.27 ± 0.47	0.99 ± 0.41	1.53 ± 0.72
	**mir210-3p**	0.81 ± 0.48	0.30 ± 0.24 ***	0.55 ± 0.35 *	0.28 ± 0.20 ***
	**mir21-5p**	2.79 ± 1.93 **	1.00 ± 0.43	2.13 ± 1.03 **	1.02 ± 0.33
	mir221-3p	1.19 ± 0.46	1.05 ± 0.63	0.87 ± 0.34	1.01 ± 0.35
	mir222-3p	1.13 ± 0.31	1.01 ± 0.52	0.83 ± 0.25	0.99 ± 0.34
	**mir23b-3p**	1.56 ± 0.46 **	1.45 ± 0.97	1.63 ± 0.70 ***	**1.61 ± 1.10 ** ^**†**^
	**mir24-2-5p**	2.04 ± 1.14 *	1.46 ± 0.91	1.48 ± 0.65	1.56 ± 0.52 *
	**mir27a-3p**	1.22 ± 0.79	0.83 ± 0.48	1.03 ± 0.41	0.81 ± 0.35 *
	mir296-5p	0.98 ± 0.55	0.80 ± 0.48	0.69 ± 0.29	0.84 ± 0.58
	**mir31-5p**	1.15 ± 0.47	0.70 ± 0.20 *	1.04 ± 0.65	0.74 ± 0.27 ***
	**mir320a**	1.11 ± 0.60	0.69 ± 0.13 **	1.11 ± 0.60	0.69 ± 0.13 *
	mir34a-3p	1.09 ± 0.58	1.24 ± 0.38	0.79 ± 0.40	1.31 ± 0.37
	**mir503-5p**	1.09 ± 0.77	0.88 ± 0.35	**0.74 ± 0.44 ** ^**†**^	0.88 ± 0.44
	mir93-5p	1.26 ± 0.39	1.04 ± 0.44	0.93 ± 0.29	1.05 ± 0.28

Fold changes printed in bold indicate that miRNAs were used for pathway analysis and are either altered by trend or significantly (^†^: *p* < 0.1; *: *p* < 0.5; **: *p* < 0.01; ***: *p* < 0.001).

**Table 2:** Pathway analysis of microRNAs that were altered by current and transient hyperglycemia (HG; HGNG) and hypoxia (HO; HONO). Pathway analysis used three software tools designed for analysis of miRNAs, i.e., DIANA miRPath, MIENTURNET, and miRPathDB. The presented data are a combination of results of the above-mentioned three softwares.

		**DIANA miRPath**	**Mienturnet**	**miRPathDB**
**Current hypoxia**	**HO normalized to HK:**			
	**Proteoglycans in cancer**	x		x
	**FOXO signalling pathway**		x	x
	**HO normalized to all angiomiRs:**			
	**Proteoglycans in cancer**	x	x	x
	**FOXO signalling pathway**		x	x
	Cell cycle	x	x	x
	HIF-1 signalling pathway		x	x
	Estrogen signalling pathway		x	x
	Ras signalling pathway		x	x
	Neurotrophin signalling pathway			
	P53 signalling pathway		x	x
	MAPK signalling pathway		x	x
	Prolactin signalling pathway		x	x
	Progesterone mediated oocyte maturation		x	x
**Transient hypoxia**	**HONO normalized to HK:**			
	**Proteoglycans in cancer**	x	x	x
	**HIF-1 signalling pathway**		x	x
	**Adherens junctions**	x	x	
	**HONO normalized to all angiomiRs:**			
	**Proteoglycans in cancer**	x	x	x
	**HIF-1 signalling pathway**		x	x
	**Adherens junctions**	x	x	
	Estrogen signalling pathway	x	x	x
	Ras signalling pathway		x	x
	FOXO signalling pathway		x	x
	Neurotrophin signalling pathway		x	x
	P53 signalling pathway	x	x	
	Protein processing in ER	x	x	

Pathways simultaneously identified when normalized to the geometric mean of housekeeping genes (HK) and when normalized to the geometric mean of all angiomiRs, are printed in bold.

**Table 3:** Primers for angiomiRs and housekeeping RNAs.

miRNA	Qiagen Primer Assay ID	Reference
**AngiomiRs:**		
let-7a-2-3p	Hs_let-7a-2*_2	[62]
miR-132-3p	Hs_miR-132_1	[63,64]
miR-134-5p	Hs_miR-134_2	[64]
miR-139-5p	Hs_miR-139_1	[65]
miR-181b-5p	Hs_miR-181b_1	[62]
miR-199a-3p	Hs_miR-199a-3p_1	[66]
miR-20a-5p	Hs_miR-20a_1	[67,68]
miR-210-3p	Hs_miR-210_1	[68]
miR-21-5p	Hs_miR-21_2	[69]
miR-221-3p	Hs_miR-221_1	[70]
miR-222-3p	Hs_miR-222_2	[70]
miR-23b-3p	Hs_miR-23b_2	[71]
miR-24-2-5p	Hs_miR-24-2*_1	[71]
miR-27a-3p	Hs_miR-27a_1	[71]
miR-296-5p	Hs_miR-296-5p_1	[72]
miR-31-5p	Hs_miR-31_1	[73]
miR-320a	Hs_miR-320a_1	[74]
miR-34a_3p	Hs_miR-34a*_1	[62]
miR-503-5p	Hs_miR-503_2	[71]
miR-93-5p	Hs_miR-93_1	[72]
**Housekeeping RNAs:**		
RNU6-6P	Hs_RNU6-2_11	[60]
miR-191-5p	Hs_miR-191_1	[59,61]
mir-28-3p	Hs_miR-28-3p_1	[59,61]
mir-30b	Hs_miR-30b_1	[58]
miR-423-3p	Hs_miR_423_1	[58]

## Data Availability

The data presented in this study are available on request from the corresponding author.

## References

[1] Tisi D.K., Burns D.H., Luskey G.W., Koski K.G. (2011). Fetal exposure to altered amniotic fluid glucose, insulin, and insulin-like growth factor-binding protein 1 occurs before screening for gestational diabetes mellitus. Diabetes Care.

[2] Escobar J., Teramo K., Stefanovic V., Andersson S., Asensi M.A., Arduini A., Cubells E., Sastre J., Vento M. (2013). Amniotic fluid oxidative and nitrosative stress biomarkers correlate with fetal chronic hypoxia in diabetic pregnancies. Neonatology.

[3] Taricco E., Radaelli T., Rossi G., Nobile de Santis M.S., Bulfamante G.P., Avagliano L., Cetin I. (2009). Effects of gestational diabetes on fetal oxygen and glucose levels in vivo. BJOG.

[4] Teramo K.A., Hiilesmaa V.K., Schwartz R., Clemons G.K., Widness J.A. (2004). Amniotic fluid and cord plasma erythropoietin levels in pregnancies complicated by preeclampsia, pregnancy-induced hypertension and chronic hypertension. J. Perinat. Med..

[5] Salafia C.M., Minior V.K., Lopez-Zeno J.A., Whittington S.S., Pezzullo J.C., Vintzileos A.M. (1995). Relationship between placental histologic features and umbilical cord blood gases in preterm gestations. Am. J. Obstet. Gynecol..

[6] Giacco F., Brownlee M. (2010). Oxidative stress and diabetic complications. Circ. Res..

[7] Bakker W., Eringa E.C., Sipkema P., van Hinsbergh V.W. (2009). Endothelial dysfunction and diabetes: Roles of hyperglycemia, impaired insulin signaling and obesity. Cell Tissue Res..

[8] Mochol J., Gawrys J., Gajecki D., Szahidewicz-Krupska E., Martynowicz H., Doroszko A. (2021). Cardiovascular Disorders Triggered by Obstructive Sleep Apnea-A Focus on Endothelium and Blood Components. Int. J. Mol. Sci..

[9] Song Y., Xing H., He Y., Zhang Z., Shi G., Wu S., Liu Y., Harrington E.O., Sellke F.W., Feng J. (2021). Inhibition of mitochondrial reactive oxygen species improves coronary endothelial function after cardioplegic hypoxia/reoxygenation. J. Thorac. Cardiovasc. Surg..

[10] Frost A.L., Suriano K., Aye C.Y.L., Leeson P., Lewandowski A.J. (2021). The Immediate and Long-Term Impact of Preeclampsia on Offspring Vascular and Cardiac Physiology in the Preterm Infant. Front. Pediatr..

[11] Sallam N.A., Palmgren V.A.C., Singh R.D., John C.M., Thompson J.A. (2018). Programming of Vascular Dysfunction in the Intrauterine Milieu of Diabetic Pregnancies. Int. J. Mol. Sci..

[12] Cvitic S., Novakovic B., Gordon L., Ulz C.M., Muhlberger M., Diaz-Perez F.I., Joo J.E., Svendova V., Schimek M.G., Trajanoski S. (2018). Human fetoplacental arterial and venous endothelial cells are differentially programmed by gestational diabetes mellitus, resulting in cell-specific barrier function changes. Diabetologia.

[13] Brodowski L., Zindler T., von Hardenberg S., Schroder-Heurich B., von Kaisenberg C.S., Frieling H., Hubel C.A., Dork T., von Versen-Hoynck F. (2019). Preeclampsia-Associated Alteration of DNA Methylation in Fetal Endothelial Progenitor Cells. Front. Cell Dev. Biol..

[14] King G.L., Kunisaki M., Nishio Y., Inoguchi T., Shiba T., Xia P. (1996). Biochemical and molecular mechanisms in the development of diabetic vascular complications. Diabetes.

[15] Fioretto P., Kim Y., Mauer M. (1998). Diabetic nephropathy as a model of reversibility of established renal lesions. Curr. Opin. Nephrol. Hypertens..

[16] Pepin M.E., Schiano C., Miceli M., Benincasa G., Mansueto G., Grimaldi V., Soricelli A., Wende A.R., Napoli C. (2021). The human aortic endothelium undergoes dose-dependent DNA methylation in response to transient hyperglycemia. Exp. Cell Res..

[17] Paneni F., Mocharla P., Akhmedov A., Costantino S., Osto E., Volpe M., Luscher T.F., Cosentino F. (2012). Gene silencing of the mitochondrial adaptor p66(Shc) suppresses vascular hyperglycemic memory in diabetes. Circ. Res..

[18] El-Osta A., Brasacchio D., Yao D., Pocai A., Jones P.L., Roeder R.G., Cooper M.E., Brownlee M. (2008). Transient high glucose causes persistent epigenetic changes and altered gene expression during subsequent normoglycemia. J. Exp. Med..

[19] Brasacchio D., Okabe J., Tikellis C., Balcerczyk A., George P., Baker E.K., Calkin A.C., Brownlee M., Cooper M.E., El-Osta A. (2009). Hyperglycemia induces a dynamic cooperativity of histone methylase and demethylase enzymes associated with gene-activating epigenetic marks that coexist on the lysine tail. Diabetes.

[20] Nangaku M., Hirakawa Y., Mimura I., Inagi R., Tanaka T. (2017). Epigenetic Changes in the Acute Kidney Injury-to-Chronic Kidney Disease Transition. Nephron.

[21] Saxena K., Jolly M.K. (2019). Acute vs. Chronic vs. Cyclic Hypoxia: Their Differential Dynamics, Molecular Mechanisms, and Effects on Tumor Progression. Biomolecules.

[22] Hartel F.V., Holl M., Arshad M., Aslam M., Gunduz D., Weyand M., Micoogullari M., Abdallah Y., Piper H.M., Noll T. (2010). Transient hypoxia induces ERK-dependent anti-apoptotic cell survival in endothelial cells. Am. J. Physiol. Cell Physiol..

[23] Liu O.H., Kiema M., Beter M., Yla-Herttuala S., Laakkonen J.P., Kaikkonen M.U. (2020). Hypoxia-Mediated Regulation of Histone Demethylases Affects Angiogenesis-Associated Functions in Endothelial Cells. Arterioscler. Thromb. Vasc. Biol..

[24] Gulyaeva L.F., Kushlinskiy N.E. (2016). Regulatory mechanisms of microRNA expression. J. Transl. Med..

[25] Wu F., Yang Z., Li G. (2009). Role of specific microRNAs for endothelial function and angiogenesis. Biochem. Biophys. Res. Commun..

[26] Wang S., Olson E.N. (2009). AngiomiRs--key regulators of angiogenesis. Curr. Opin. Genet. Dev..

[27] Monticelli S., Natoli G. (2013). Short-term memory of danger signals and environmental stimuli in immune cells. Nat. Immunol..

[28] Costantino S., Paneni F., Luscher T.F., Cosentino F. (2016). MicroRNA profiling unveils hyperglycaemic memory in the diabetic heart. Eur. Heart J..

[29] Zhong X., Liao Y., Chen L., Liu G., Feng Y., Zeng T., Zhang J. (2015). The MicroRNAs in the Pathogenesis of Metabolic Memory. Endocrinology.

[30] Yuan S.Y., Breslin J.W., Perrin R., Gaudreault N., Guo M., Kargozaran H., Wu M.H. (2007). Microvascular permeability in diabetes and insulin resistance. Microcirculation.

[31] Simionescu M. (2007). Implications of early structural-functional changes in the endothelium for vascular disease. Arterioscler. Thromb. Vasc. Biol..

[32] Liu R., Guan S., Gao Z., Wang J., Xu J., Hao Z., Zhang Y., Yang S., Guo Z., Yang J. (2021). Pathological Hyperinsulinemia and Hyperglycemia in the Impaired Glucose Tolerance Stage Mediate Endothelial Dysfunction Through miR-21, PTEN/AKT/eNOS, and MARK/ET-1 Pathways. Front. Endocrinol..

[33] Rask-Madsen C., King G.L. (2013). Vascular complications of diabetes: Mechanisms of injury and protective factors. Cell Metab..

[34] Flynn M.C., Kraakman M.J., Tikellis C., Lee M.K.S., Hanssen N.M.J., Kammoun H.L., Pickering R.J., Dragoljevic D., Al-Sharea A., Barrett T.J. (2020). Transient Intermittent Hyperglycemia Accelerates Atherosclerosis by Promoting Myelopoiesis. Circ. Res..

[35] Nallamshetty S., Chan S.Y., Loscalzo J. (2013). Hypoxia: A master regulator of microRNA biogenesis and activity. Free Radic. Biol. Med..

[36] Bertero T., Rezzonico R., Pottier N., Mari B. (2017). Impact of MicroRNAs in the Cellular Response to Hypoxia. Int. Rev. Cell Mol. Biol..

[37] Greco S., Martelli F. (2014). MicroRNAs in Hypoxia Response. Antioxid. Redox Signal..

[38] Yao C., Shi X., Zhang Z., Zhou S., Qian T., Wang Y., Ding F., Gu X., Yu B. (2016). Hypoxia-Induced Upregulation of miR-132 Promotes Schwann Cell Migration After Sciatic Nerve Injury by Targeting PRKAG3. Mol. Neurobiol..

[39] Zhou C.H., Zhang X.P., Liu F., Wang W. (2015). Modeling the interplay between the HIF-1 and p53 pathways in hypoxia. Sci. Rep..

[40] Pranzini E., Leo A., Rapizzi E., Ramazzotti M., Magherini F., Giovannelli L., Caselli A., Cirri P., Taddei M.L., Paoli P. (2019). miR-210-3p mediates metabolic adaptation and sustains DNA damage repair of resistant colon cancer cells to treatment with 5-fluorouracil. Mol. Carcinog..

[41] Prado M.S.G., de Jesus M.L., de Goes T.C., Mendonca L.S.O., Kaneto C.M. (2020). Downregulation of circulating miR-320a and target gene prediction in patients with diabetic retinopathy. BMC Res. Notes.

[42] Jo S., Xu G., Jing G., Chen J., Shalev A. (2021). Human Glucagon Expression Is under the Control of miR-320a. Endocrinology.

[43] Strutz J., Cvitic S., Hackl H., Kashofer K., Appel H.M., Thuringer A., Desoye G., Koolwijk P., Hiden U. (2018). Gestational diabetes alters microRNA signatures in human feto-placental endothelial cells depending on fetal sex. Clin. Sci..

[44] He L., Wang X., Jin Y., Xu W., Guan Y., Wu J., Han S., Liu G. (2021). Identification and validation of the miRNA-mRNA regulatory network in fetoplacental arterial endothelial cells of gestational diabetes mellitus. Bioengineered.

[45] Humphries D.E., Lee S.L., Fanburg B.L., Silbert J.E. (1986). Effects of hypoxia and hyperoxia on proteoglycan production by bovine pulmonary artery endothelial cells. J. Cell. Physiol..

[46] Aslam M., Schluter K.D., Rohrbach S., Rafiq A., Nazli S., Piper H.M., Noll T., Schulz R., Gunduz D. (2013). Hypoxia-reoxygenation-induced endothelial barrier failure: Role of RhoA, Rac1 and myosin light chain kinase. J. Physiol..

[47] Wojciak-Stothard B., Tsang L.Y., Haworth S.G. (2005). Rac and Rho play opposing roles in the regulation of hypoxia/reoxygenation-induced permeability changes in pulmonary artery endothelial cells. Am. J. Physiol. Lung Cell. Mol. Physiol..

[48] Schwarzenbach H., da Silva A.M., Calin G., Pantel K. (2015). Data Normalization Strategies for MicroRNA Quantification. Clin. Chem..

[49] Leopold B., Strutz J., Weiss E., Gindlhuber J., Birner-Gruenberger R., Hackl H., Appel H.M., Cvitic S., Hiden U. (2019). Outgrowth, proliferation, viability, angiogenesis and phenotype of primary human endothelial cells in different purchasable endothelial culture media: Feed wisely. Histochem. Cell Biol..

[50] Lang I., Schweizer A., Hiden U., Ghaffari-Tabrizi N., Hagendorfer G., Bilban M., Pabst M.A., Korgun E.T., Dohr G., Desoye G. (2008). Human fetal placental endothelial cells have a mature arterial and a juvenile venous phenotype with adipogenic and osteogenic differentiation potential. Differentiation.

[51] Arikan G.M., Scholz H.S., Petru E., Haeusler M.C., Haas J., Weiss P.A. (2000). Cord blood oxygen saturation in vigorous infants at birth: What is normal?. BJOG.

[52] Livak K.J., Schmittgen T.D. (2001). Analysis of relative gene expression data using real-time quantitative PCR and the 2(-Delta Delta C(T)) Method. Methods.

[53] Babion I., Snoek B.C., van de Wiel M.A., Wilting S.M., Steenbergen R.D.M. (2017). A Strategy to Find Suitable Reference Genes for miRNA Quantitative PCR Analysis and Its Application to Cervical Specimens. J. Mol. Diagn..

[54] Wotschofsky Z., Meyer H.A., Jung M., Fendler A., Wagner I., Stephan C., Busch J., Erbersdobler A., Disch A.C., Mollenkopf H.J. (2011). Reference genes for the relative quantification of microRNAs in renal cell carcinomas and their metastases. Anal. Biochem..

[55] Marabita F., de Candia P., Torri A., Tegner J., Abrignani S., Rossi R.L. (2016). Normalization of circulating microRNA expression data obtained by quantitative real-time RT-PCR. Brief. Bioinform..

[56] Rice J., Roberts H., Rai S.N., Galandiuk S. (2015). Housekeeping genes for studies of plasma microRNA: A need for more precise standardization. Surgery.

[57] Hijmans J.G., Stockelman K., Levy M., Brewster L.M., Bammert T.D., Greiner J.J., Connick E., DeSouza C.A. (2019). Effects of HIV-1 gp120 and TAT-derived microvesicles on endothelial cell function. J. Appl. Physiol..

[58] Anand S., Majeti B.K., Acevedo L.M., Murphy E.A., Mukthavaram R., Scheppke L., Huang M., Shields D.J., Lindquist J.N., Lapinski P.E. (2010). MicroRNA-132-mediated loss of p120RasGAP activates the endothelium to facilitate pathological angiogenesis. Nat. Med..

[59] Wang H.W., Su S.H., Wang Y.L., Chang S.T., Liao K.H., Lo H.H., Chiu Y.L., Hsieh T.H., Huang T.S., Lin C.S. (2016). MicroRNA-134 Contributes to Glucose-Induced Endothelial Cell Dysfunction and This Effect Can Be Reversed by Far-Infrared Irradiation. PLoS ONE.

[60] Walz J.M., Wecker T., Zhang P.P., Cakir B., Gruening B., Agostini H., Reuer T., Ludwig F., Boneva S., Faerber L. (2019). Impact of angiogenic activation and inhibition on miRNA profiles of human retinal endothelial cells. Exp. Eye Res..

[61] Wang L., Liu W.X., Huang X.G. (2020). MicroRNA-199a-3p inhibits angiogenesis by targeting the VEGF/PI3K/AKT signalling pathway in an in vitro model of diabetic retinopathy. Exp. Mol. Pathol..

[62] Guo Y., Du F., Tan Y.L., Luo J., Xiong D., Song W.T. (2021). VEGF-mediated angiogenesis in retinopathy of prematurity is co-regulated by miR-17-5p and miR-20a-5p. Biochem. Cell Biol..

[63] Mirzaei Bavil F., Karimi-Sales E., Alihemmati A., Alipour M.R. (2019). Effect of ghrelin on hypoxia-related cardiac angiogenesis: Involvement of miR-210 signalling pathway. Arch. Physiol. Biochem..

[64] Penaloza E., Soto-Carrasco G., Krause B.J. (2020). MiR-21-5p directly contributes to regulating eNOS expression in human artery endothelial cells under normoxia and hypoxia. Biochem. Pharmacol..

[65] Poliseno L., Tuccoli A., Mariani L., Evangelista M., Citti L., Woods K., Mercatanti A., Hammond S., Rainaldi G. (2006). MicroRNAs modulate the angiogenic properties of HUVECs. Blood.

[66] Zhou Q., Gallagher R., Ufret-Vincenty R., Li X., Olson E.N., Wang S. (2011). Regulation of angiogenesis and choroidal neovascularization by members of microRNA-23~27~24 clusters. Proc. Natl. Acad. Sci. USA.

[67] Jia L., Zhou X., Huang X., Xu X., Jia Y., Wu Y., Yao J., Wu Y., Wang K. (2018). Maternal and umbilical cord serum-derived exosomes enhance endothelial cell proliferation and migration. FASEB J..

[68] Huang J., Yu M., Yin W., Liang B., Li A., Li J., Li X., Zhao S., Liu F. (2021). Development of a novel RNAi therapy: Engineered miR-31 exosomes promoted the healing of diabetic wounds. Bioact. Mater..

[69] Zampetaki A., Willeit P., Burr S., Yin X., Langley S.R., Kiechl S., Klein R., Rossing P., Chaturvedi N., Mayr M. (2016). Angiogenic microRNAs Linked to Incidence and Progression of Diabetic Retinopathy in Type 1 Diabetes. Diabetes.

[70] Popov T.M., Giragosyan S., Petkova V., Stancheva G., Marinov T., Belitova M., Rangachev J., Popova D., Kaneva R.P. (2020). Proangiogenic signature in advanced laryngeal carcinoma after microRNA expression profiling. Mol. Biol. Rep..

[71] Liang L., Zhao L., Zan Y., Zhu Q., Ren J., Zhao X. (2017). MiR-93-5p enhances growth and angiogenesis capacity of HUVECs by down-regulating EPLIN. Oncotarget.

[72] Vlachos I.S., Zagganas K., Paraskevopoulou M.D., Georgakilas G., Karagkouni D., Vergoulis T., Dalamagas T., Hatzigeorgiou A.G. (2015). DIANA-miRPath v3.0: Deciphering microRNA function with experimental support. Nucleic Acids Res..

[73] Licursi V., Conte F., Fiscon G., Paci P. (2019). MIENTURNET: An interactive web tool for microRNA-target enrichment and network-based analysis. BMC Bioinform..

[74] Kehl T., Kern F., Backes C., Fehlmann T., Stockel D., Meese E., Lenhof H.P., Keller A. (2020). miRPathDB 2.0: A novel release of the miRNA Pathway Dictionary Database. Nucleic Acids Res..

